# 

**Published:** 1862-01

**Authors:** 


### CONTENTS.---JANUARY, 1862.

### ORIGINAL CONTRIBUTIONS.

PAGE

ART. I. OVARIAN TUMORS. By WM. H. BYFORD, A. M., M. D., Professor of Obstetrics and Diseases of Women and Children, in the Medical Department, Lind University, Chicago, Ill.,

2d Paper:—Anatomy, Nature and Pathology of Ovarian Tumors,........ 1

ART. II. A Case of Complex Labor, Double Fissure of the Os Uteri, with Cicatrices; Retained Placenta, with Hour-glass Contractions. By CHAS. G. SMITH, M. D., of Chicago, Ill........ 13

ART. III. Case of Fracture of the Skull. By O. B. ORMSBY, M. D., Assistant Surgeon of the 18th Regiment, I. V.,........ 16

ART. IV. New Operation for Obstinate Strabismus. By E. ANDREWS, A. M., M.D., Prof. of Surg. in Med. Dep. of Lind Univ., and Surgeon of Mercy Hospital,........ 20

### CORRESPONDENCE.

Congenital Malformation—Extract of Belladonna in Mammary Inflammation. By J. J. LESCHER, M. D., Mt. Carmel, Ills........ 22

Improvement upon the Adhesive-Strap Counter-Extension. By Prof. E. ANDREWS,........ 26

### PROCEEDINGS OF SOCIETIES.

Chicago Medical Society—Regular Meeting January 21, 1862. Discussion on the Nature and Treatment of Constipation........ 28

### SELECTIONS.

Per-chloride of Iron in Erysipelas,........ 31

Pyæmia and Hospital Gangrene. By Prof. JUNGKEN, of Berlin,........ 35

Uterine Hæmatocele. By CHAS. WEST, M. D., London, England,........ 39

Nature in the Cure of Disease,........ 45

A New Method of Treating Dry Labors,........ 47

### BOOK NOTICES.

The Placenta, the Organic Nervous System, the Blood, the Oxygen, and the Animal Nervous System, Physiologically Examined. By JOHN O'REILLY, M. D., etc., etc.,........ 49

Address Delivered at the Opening of the New Clinical Lecture Room of the Philadelphia Hospital. By Dr. J. L. LUDLOW, one of the Medical Examining Board, etc.,........ 50

Medical Jurisprudence. By ALFRED SWAYNE TAYLOR, M. D., F. R. S., Fellow of the Royal College of Physicians, etc., etc........ 52

BRAITHWAITE'S Retrospect. Part XLIV........ 53

### EDITORIAL.

Local Medical Societies,........ 54

Prof. TITUS DEVILLE,........ 54

Rush Medical College Commencement,........ 55

Medical Department of Lind University,........ 56

Books Received,—Wanted,—Apology,........ 56

SPECIAL SELECTIONS: Chlorate of Potash as a Remedy for Fœtid Breath,........ 56

Syphilo-Vaccination,........ 57

Sulphuret of Lime in Regeneration of Bone,........ 58

Leucorrhœa in Pregnant Women,........ 57

New Method of Administering Chloroform,........ 58

Phthisis,........ 58

Reproduction of the Inferior Maxillary,........ 58

Quinine in the Dropsy of Scarlatina,........ 59

New Procedure for the Ligature of the Superficial Palmar Arch,........ 60

New Surgical Propositions,........ 61

Clergyman's Sore Throat,........ 62

Position in Etherization,........ 63

